# Preliminary characterization of the probiotic properties of *Candida famata* and *Geobacillus thermoleovorans*

**Published:** 2011-09

**Authors:** A Mahdhi, Z Hmila, A Behi, A Bakhrouf

**Affiliations:** Laboratory of Analysis, Treatment and Valorization of Pollutants of the Environment and Products (LATVPEP). Faculty of Pharmacy, University of Monastir, 5000. Tunisia.

**Keywords:** Yeast, Bacteria, Probiotic, Exoenzyme, Adhesion

## Abstract

**Background and Objective:**

Probiotics are live microbial feed supplements which beneficially affect the host animal by improving its intestinal microbial balance, producing metabolites which inhibit the colonization or growth of other microorganisms or by competing with them for resources such as nutrients or space. The aim of this study was to investigate the probiotic properties of *Candida famata* and *Geobacillus thermoleovorans*.

**Material and Methods:**

In this study, yeast and bacterial strains isolated from pure oil waste were identified using Api 50 CHB and Api Candida Systems and their probiotic properties were studied through antimicrobial activity, biofilm production, adherence assay and enzymatic characterization.

**Results and Conclusion:**

According to biochemical analyses, these strains corresponded to *Geobacillus thermoleovorans* and *Candida famata*. Antagonism assay results showed that the tested strains have an inhibitory effect against tested pathogenic bacteria. The yeast *Candida famata* was unable to produce biofilm on Congo Red Agar (CRA), while the bacterial strain was a slime producer. Adherence assays to abiotic surfaces revealed that the investigated strains were fairly adhesive to polystyrene with values ranging from 0.18 to 0.34 at 595 nm. The enzymatic characterization revealed that the tested strains expressed enzymes such as phosphatase alkaline, esterase lipase (C8), amylase, lipase, lecitenase and caseinase. The obtained results may allow the isolated strains to be considered as having the potential to be candidate probiotics.

## INTRODUCTION

Probiotics are live microbial feed supplements which beneficially affect the host animal by improving its intestinal microbial balance (1). The potential benefits that are claimed include improved nutrition and growth and prevention of various gastrointestinal disorders. Probiotic-containing products are available for animal and human nutrition (2). The use of probiotics to enhance intestinal health has been proposed for many years (3, 4). As recently revisited by the Joint Food and Agriculture Organization/World Health Organization Expert Consultation on Evaluation of Health and Nutritional Properties of Probiotics in Food Including Powder Milk with live lactic acid bacteria, probiotic strains are defined as live micro-organisms that, when consumed in an adequate amount as part of food, confer a health benefit on the host (4). Probiotic strains are considered non-pathogenic and safe. Many mechanisms have been postulated by which probiotics could enhance intestinal health, including competition for limited nutrients, inhibition of the epithelial and mucosal adherence of pathogens, inhibition of epithelial invasion by pathogens, the production of antimicrobial substances and/or the stimulation of mucosal immunity. A group of antibacterial proteins known as bacteriocins, produced by Gram-positive bacteria including lactobacilli, has been shown to display a wide antibacterial spectrum against Gram-positive bacteria (5). Several studies demonstrated that probiotics enhance growth performance, survival, immunity, and disease resistance (6–8). Marques et al. (6) and Mahdhi et al., (7) demonstrated that Bacillus spp and Candida utilis provide protection against pathogenic Vibrio and can be considered as potential candidate probiotics. This study aimed to investigate the probiotic properties of Candida famata and Geobacillus thermoleovorans strains through the inhibition ability of pathogens, biofilm formation and enzymatic characterization in order to find potential probiotic strains for animal and human uses.

## MATERIALS AND METHODS

**Bacterial strains**. Bacterial strains were isolated aseptically from pure oil waste. Gram and catalase positive rods were retained. These were identified using standard morphological and physiological techniques. We used API 50 CHB and Api 20 E (BioMérieux, Marcy-l'Etoile, France) for the identification of the bacterial strain. Yeast strain was identified using Api Candida system (BioMérieux, Marcy-l'Etoile, France). Results were read using an automated microbiological mini-API (BioMérieux, Marcy l'Etoile, France). Strains were stored at -80 °C and we routinely checked its purity during this investigation prior to use (9). Four ATCC reference strains, Vibrio parahaemolyticus (ATCC 17802), Vibrio alginolyticus (ATCC 17749), Salmonella typhimurium (ATCC 1408), and Escherichia coli (ATCC 35218) were used to study antibacterial activity.

**Well diffusion agar assay (WDAA)**. Potential probiotic strains were tested for their antagonistic activity using the well diffusion agar assay (WDAA) (5) against target strains: V. parahaemolyticus (ATCC 17802), V. alginolyticus (ATCC 17749), Salmonella typhimurium (ATCC 1408), and E. coli (ATCC 35218). The pathogenic bacteria were grown in 10 ml of nutrient broth and cultured for 24 hours on nutrient agar at 30°C. The common colonies from pure culture were suspended in 10 ml of physiological medium and well mixed for 5 min. One ml was spread over the agar plates. Potential probiotic strains, Candida famata and Geobacillus thermoleovorans, were cultured in 10 ml of Sabouraud and nutrient broth for 24 hours, 100 l of the supernatant were introduced into the wells of the MH agar medium and incubated for a period of 24 h at 30 °C. Antibacterial activity was defined as the diameter (mm) of the clear inhibitory zone formed around the well (5).

**Phenotypic characterization of slime-producing bacteria**. Qualitative detection of biofilm formation was studied using Congo red agar (CRA) methods as previously described (10). The tested strains were inoculated into the surface of CRA plates, prepared by mixing 0.8 g Congo red with 36 g saccharose (Sigma) in 1 L of brain heart infusion agar, and were incubated for 24 h at 30°C under aerobic conditions and followed overnight at room temperature. Slime producing bacteria appeared as black colonies, whereas non-slime producers remained non-pigmented (10).

**Quantitative adherence assay**. Biofilm production by probiotic strains was determined using a semi-quantitative adherence assay on 96-well tissue culture plates, as described previously (7). Strains were grown in Trypticase Soy broth supplemented with 1% (w/v) NaCl (TSB 1%, Pronadisa, Spain). Following overnight incubation at 30°C, optical density at 600 nm (OD*600*) of the bacteria was measured. An overnight culture, grown in TSB 1% at 30°C, was diluted to 1:100 in TSB supplement with 2% (w/v) glucose. A total of 200 l of cell suspension was transferred to a Ubottomed 96-well microtiter plate (Nunc, Roskilde, Denmark).

Each strain was tested in triplicate. Wells with sterile TSB alone served as controls. The plates were incubated aerobically at 30°C for 24 h. The cultures were removed and the microtiter wells were washed twice with phosphate-buffered saline (7 mM Na*2*HPO*4*, 3 mM NaH*2*PO*4* and 130 mM NaCl at pH 7.4) to remove non-adherent cells and dried in an inverted position. Adherent bacteria were fixed with 95% ethanol and stained with 100 l of 1% crystal violet (Merck, France) for 5 min. The excess stain was rinsed and poured off and the wells were washed three times with 300 l of sterile distilled water. The water was then cleared and the microplates were air-dried. The optical density of each well was measured at 570 nm (OD*570*) using an automated Multiskan reader (GIO. DE VITA E C, Rome, Italy). Biofilm formation was interpreted as highly positive (OD570 = 1), low grade positive (0.1 = OD*570* < 1), or negative (OD*570* < 0.1) (11).

**Enzymatic characterization**. Enzymatic charac-terization of the potential probiotic strains were studied with the API Zym System containing 19 substrates according to the manufacturer′s instructions (Bio-Mérieux). The activities of four other enzymes were determined following inoculation of cultures onto TSA-1 to which the following substrates were added: 0.2% starch for amylase, 1% skim milk for caseinase, 1% Tween 80 for lipase, and 5% egg yolk for phospholipase (lecithinase) activities (12). After 24h of incubation at 44°C and 37°C for the bacterial and yeast strain respectively, results were read according to the manufacturer's instructions. A positive reaction of amylase, lipase, caseinase and lecithinase is highlighted by the appearance of an aureole around the colonies.

## RESULTS

**Bacterial strain identification and Antimicrobial activity**. Bacterial strains isolated from pure oil waste were identified as Geobacillus thermoleovorans (S1) and Candida famata (S2). Potential probiotic strains exhibited greater inhibitory activity against pathogenic strains used in this study (Table 1). The inhibitory zones were about 12–20 mm in diameter.

**Table 1:** Antibacterial activity of the tested strains.

Strains	Pathogens				

	*S. typhimurium ATCC 1408*	* E. coli ATCC 35218 *	* V.parahemolitycus ATCC 17802 *	*V.parahaemolyticus ATCC 17749*	* S. aureus ATCC 25923 *
S1	6 ± 1.4	14.5 ± 2.1	15 ± 1.4	16.5 ± 4.9	17 ± 4.2
S2	12 ± 0.7	13.5 ± 2.1	0	12.5 ± 2.1	12.5 ± 0.7

S1: *Geobacillus thermoleovorans*; S2: *Candida famata;* For each average, the respective standard deviation is added (mean ± S.D).

**Adherence assay and enzymatic characterization**. The results of adherence assay showed that these strains were fairly adhesive with a values ranging from 0.18 to 0.34 at 595 nm (Table 2). Our results showed that strains S1 and S2 expressed the following enzymatic activities: phosphatase alkaline, esterase lipase (C8), amylase, lipase, lecitenase and caseinase (Table 3).

**Table 2:** Biofilm production and adherence assays.

Strains	Biofilm production	Phenotype on CRA	Adherence to polystyrene
S1	P	black	0.34 ± 0.28
S2	NP	Orange	0.18 ± 0.05

S1: *Geobacillus thermoleovorans*; S2: *Candida famata;* P: Producer; NP: No Producer; OD_570_ = 1: highly adherent, 0.1 = OD_570_ < 1: fairly adherent, OD_570_< 0.1: slightly adherent.

**Table 3:** Api ZYM enzymatic profiles of the tested strains.

Enzymes	S1	S2
Phosphatase alcaline	+	+
Esterase(C4)	+	+
Esterase Lipase(C8)	+	+
Lipase(C14)	+	+
Leucine arylamidase	+	+
Valine arylamidase	-	-
Cystine arylamidase	+	-
Trypsine	-	-
a-chymotrypsine	-	-
Phosphatase acide	+	+
Naphtol-AS-BI-phosphohydrolase	+	+
a-galactosidase	+	-
-galactosidase	+	-
-glucuronidase	-	-
a-glucosidase	+	+
-glucosidase	+	+
N-acetyl--glucosaminidase	-	-
a-mannosidase	+	+
a-fucisidase	+	+
Amylase	+	+
lecithinase	+	-
caseinase	+	-
Lipase	+	+

S1: *Geobacillus thermoleovorans*; S2: *Candida famata;* +: positive reaction; -: negative reaction.

## DISCUSSION

The present study confirms that the tested strains can be considered as potential candidate probiotics. In fact, antagonism assay revealed that these strains have an inhibitory effect against pathogenic bacteria (diameter of inhibition zone ranged from 12 to 18 mm). This demonstrates the production of antibacterial compounds that diffused through the agar inhibiting the growth of pathogens (13). Several studies suggested that the inhibitory effects of the used probiotics might be due also to the alteration of the growth medium pH or the volatile compounds production (14). In this study, the inhibitory mechanism of the interaction was not characterized, but several previously researches reported that Bacillus produces polypeptide antibiotics, such as bacitracin, gramicidin S, polymyxin, and tyrotricidin, which are active against a wide range of Gram-positive and Gram-negative bacteria (15). Furthermore, bacteriocins have been identified in Geobacillus stearothermophilus and Geobacillus thermoleovorans (16). In addition to the inhibitory ability, the efficacy of a probiotic application depends on many factors such as application level, frequency of application and environmental conditions (17). The adherence ability to abiotic surface partly explain the observed positive effect in this study. This property might help probiotic strains to persist in the gut for several days and be active during intestinal transit, participate in digestion processes, elimination of potential pathogens, and creation of a healthy environment (7).

Several microbiological studies have demonstrated the beneficial effect of probiotic bacterial and yeast strains. Indeed, it was reported that a number of bacteria such us Bacillus spp and Aeromonas hydrophila and yeasts such us Saccharomyces baulardi, C. famata and C. parapilosis, enhance protection against a pathogenic bacteria and have a significant technological benefits as starter for the production of traditional dry fermented sausages and contribute significantly to the flavor of fermented meat products and meat-flavored products (6, 18, 19). These yeasts are known to secrete lipases and/or proteases which contribute to flavor by offsetting and modifying the acidic pH produced by mixed bacterial starter culture activities through the degradation of lipids to produce free fatty acids and glycerol and the breakdown of nitrogenous compounds to amino acids with release of ammonia. Naima et al. (20), demonstrated that Candida famata has an antagonistic activity against Penicillium digitatum Sacc., agent of the green mould. Other experimental results demonstrated that potential probiotic Pseudomonas stutzeri and Candida utilis have an antibacterial activity against pathogenic bacteria and fairly adhesive to abiotic surface (7, 21). Pioneering studies by Reid and co-workers (22) have demonstrated experimentally that selected Lactobacillus strains of urovaginal origin have adhesive properties that enable them to inhibit and/or prevent the colonization of uroepithelial cells by uropathogens. The same mechanism of action has subsequently been proposed for Lactobacillus strains of intestinal origin. In vitro experimental assays have demonstrated that selected lactic acid strains are effective against diarrhoeagenic bacteria by producing metabolites such as acetic and lactic acids which decrease the pH and then inhibit the growth of bacterial pathogens (23). Moriarty (24) reported the beneficial effects of administering probiotics as a food supplement or as an additive. Probiotic strains secrete many exoenzymes and are often antagonistic against other micro-organisms, including fish and shellfish pathogenic bacteria (25). It has been documented that Bacillus bacteria and yeast such us Candida famata secrete many exoenzymes, such as proteases and lipases and these microorganisms have been used widely as putative probiotics and have a technological properties such as lipase and protease activity and growth under different environmental conditions (21, 24, 26, 27). Moreover, it has been reported that extremophiles bacteria like G. thermoleovorans are able to produce highly stable enzymes and proteins adaptable to industrial, biotechnological and bioremediation applications (16, 28). These two criteria associated to their fairly adherence to abiotic surface might improve its efficacy and explain the positive effect against pathogenic bacteria. Based on the preliminary results found in the current study, the isolated strains can be considered as a potential candidate probiotic. More studies are required to clarify their exact inhibitory mechanism.
